# Evaluation of the acute toxicity, phototoxicity and embryotoxicity of a residual aqueous fraction from extract of the Antarctic moss *Sanionia uncinata*

**DOI:** 10.1186/s40360-019-0353-3

**Published:** 2019-12-19

**Authors:** Andréia da Silva Fernandes, Lara Barroso Brito, Gisele Augusto Rodrigues Oliveira, Elisa Raquel Anastácio Ferraz, Heitor Evangelista, José Luiz Mazzei, Israel Felzenszwalb

**Affiliations:** 1 Laboratory of Environmental Mutagenesis, Department of Biophysics and Biometry, University of the State of Rio de Janeiro, Rio de Janeiro, RJ Brazil; 2 Environmental Toxicology Research Laboratory (EnvTox), Faculty of Pharmacy, Federal University of Goiás (UFG), Goiânia, GO Brazil; 3 Laboratory of Toxicology, Department of Pharmacy and Pharmaceutical Administration, Pharmacy College, Fluminense Federal University, Niteroi, RJ Brazil; 4 Laboratory of Radioecology and Global Changes, Department of Biophysics and Biometry, University of the State of Rio de Janeiro, Rio de Janeiro, RJ Brazil; 5 Department of Natural Products, Institute of Drug Technology, Oswaldo Cruz Foundation, Rio de Janeiro, RJ Brazil

**Keywords:** *Sanionia uncinata*, Sun protection factor, Embryotoxicity, Cell death, 3D cell, Phototoxicity

## Abstract

**Background:**

Ultraviolet (UV) radiation is the main exogenous inductor of skin damage and so photoprotection is important to control skin disorders. The Antarctic moss *Sanionia uncinata* is an important source of antioxidants and the photoprotective activity of its organic extracts has been investigated. This study aimed to evaluate the potential photoprotection, cytotoxicity and embryotoxicity of residual aqueous fraction (AF) from the moss *S. uncinata*.

**Methods:**

UV-visible spectrum and SPF (sun protection factor) were determined by spectrophotometry. Embryotoxicity potential was evaluated by Fish embryo-larval toxicity test using zebrafish (*Danio rerio*) as organism model. Cell death assays by water-soluble tetrazolium salt (WST-1) and lactate dehydrogenase (LDH) were investigated using HaCaT keratinocyte cell line cultured in monolayers and three dimensions (3D). Phototoxicity and association with UV-filters were performed by 3T3 neutral red uptake test.

**Results:**

The AF showed sharp absorption bands in the UV region and less pronounced in the visible region. The SPF was low (2.5 ± 0.3), but the SPF values of benzophenone-3 and octyl-methoxycinnamate increased ~ 3 and 4 times more, respectively, in association with AF. The AF did not induce significant lethal and sublethal effects on zebrafish early-life stages. In monolayers, the HaCaT cell viability, evaluated by WST-1, was above 70% by ≤0.4 mg AF/mL after 48 and 72-h exposure, whereas ≤1 mg AF/mL after 24-h exposure. The LDH assay showed that the cell viability was above 70% by ≤0.4 mg AF/mL even after 72-h exposure, but ≤1 mg/mL after 24 and 48-h exposure. In 3D cell culture, an increased cell resistance to toxicity was observed, because cell viability of HaCaT cell by WST-1 and LDH was above ~ 90% when using ≤1 and 4 mg AF/mL, respectively. The AF demonstrated values of photo irritation factor < 2 and of photo effect < 0.1, even though in association with UV-filters.

**Conclusions:**

The residual AF absorbs UV-vis spectrum, increased SPF values of BP-3 and OMC and does not induce embryotoxicity to zebrafish early life-stage. The cell death assays allowed establishing non-toxic doses of AF and phototoxicity was not detected. AF of *S. uncinata* presents a good potential for skin photoprotection against UV-radiation.

## Background

The human incidences of skin cancer and photoaging, that result from the excessive solar ultraviolet radiation (UVR) exposure, are increasing around the world [1]. The UVR is classified into UVA (from 315 to 400 nm), UVB (from 280 to 315 nm) and UVC (from 100 to 280 nm) [2]. Acute and chronic skin exposures to UVR result in deleterious effects on human skin such as erythema, photo-ageing and initiation of carcinogenic processes [3]. In general, sunscreens are intended to protect the surface of the skin by reflecting (inorganic UV filters) or absorbing (organic UV filters) radiation [4]. However, recent publications have shown that more commonly used topical sunscreens do not provide full protection, failing to act on relevant biochemical events, as degradation of the extracellular matrix, immunosuppression, inhibition on the release of reactive oxygen species (ROS) and of reactive nitrogen species [5, 6]. Moreover, some UV-filters can diffuse into deep layers of the skin and the systemic absorption can result in efficiency loss and local and systemic toxicity [7].

The use of natural antioxidant agents, such as phenolic compounds especially flavonoids present in plant kingdom, might be an effective strategy for minimize the deleterious effects of UV-induced reactive species. These compounds have been used in topical cosmetic formulations by the pharmaceutical industry [8], because flavonoids are structurally similar to chemical filters which makes it susceptible to absorption of UVR. The association of natural products with sunscreen products can extend the benefits of solar protection by offering additional features that minimize damage in tissues chronically exposed to UVR [9].

The Antarctica moss *Sanionia uncinata* (Hedw.) Loeske from King George Island is directly exposed to high levels of solar radiation during the summer (December to late March), when soil and air temperatures typically remain above freezing. At sub-Antarctic islands such as the collection site, UVR levels reaching the surface are about four times higher in the summer (in terms of maximum daily UV index) than in the winter or autumn [10]. The influence of the Antarctic ozone hole on surface climate is most pronounced during the austral summer season [11]. Due to this, Antarctic *S. uncinata* has been exposed to greater solar UVR and consequently it has tolerated and acclimatized well to that singular environmental stresses, possibly via increase in the synthesis of important constituents for protecting, such as flavonoids [12].

Our research group has showed that aqueous and hydroethanolic (HE) extracts from *S. uncinata* act as protectors against plasmid DNA cleavage by way of scavenging of superoxide radical anion and hydroxyl radical [10], and do not induce point mutations [13]. Nevertheless, these extracts induced an increase in DNA cleavage via a Fenton-like reaction [10]. We also demonstrated that the inhibition of ROS-damage by *S. uncinata* has been associated with the flavonoid constituents such as flavone, flavanone, flavonols, chacones and catechin [14]. We also demonstrated that HE, ethanolic (EE) and methanolic (ME) extracts showed high sun protection factor (SPF) and enhanced SPF of benzophenone-3 (BP-3), indicating that their constituents could be considered as attractive candidates for protection against UV-induced erythema formation. Besides, HE, EE and ME did not induce photomutation and showed photoprotection against the photobiological and ROS-inducing effects of the UVA radiation [14].

Since *S. uncinata* extracts, principally HE, present potential photoprotection against UV damage and, consequently have been considered as an attractive candidate for cosmetic and dermatological applications, the present study aimed to evaluate the potential photoprotection, cytotoxicity and embryotoxicity of an aqueous fraction (AF) from HE of the polar moss *S. uncinata*.

## Methods

### Moss material and liquid-liquid partitioning

Samples of *S. uncinata* were collected in the vicinity of the Brazilian Comandante Ferraz Antarctic Station (62°05′S, 58°24′W) in King George Island, located in the South Shetland Islands, Antarctic Peninsula in January 2012, using aseptic procedures, storing in plastic bags and keeping frozen until being processed in laboratory conditions. The specimens were identified by Prof. Dr. Antônio Batista Pereira from the Federal University of Pampa, Rio Grande do Sul (Brazil), and a voucher specimen was deposited in the Herbarium of the State University of Rio de Janeiro, Brazil, under the registration number H-RJ 11,811. The collection of sample material was authorized by the Brazilian Ministry of the Environment.

Preparation of the raw hydroethanolic extract was previously described in detail by our group [14]. A portion (1 g) of the dried hydroethanolic extract was solubilized in 30 mL of water: methanol solution (7,3) and was fractionated by sequential liquid-liquid partition (10 mL each solvent) with *n-*hexane (Hf), dichloromethane (Df), ethyl acetate (Ef), *n-*butanol (Bf), remaining the residual aqueous fraction (AF). The extractive solvents were removed using a rotary evaporator under reduced pressure at 40 °C and the fractions were frozen and lyophilized to remove the remaining water. The yields (w/w) of dried partition fractions were 6.13, 8.63, 7.50, 22.33 and 62.43%, respectively, for Hf, Df, Ef, Bf and AF.

### UV-visible spectrophotometry

An aliquot of 200 μL of AF solution (0.25 mg/mL) in isopropanol were transferred to a 96-well microplate and the absorption spectrum was read at 10 nm intervals from 200 to 800 nm using a microplate reader (μ-Quant, Biotek, USA).

### In vitro sun protection factor

In vitro SPF was determined and calculated according to previously described protocols [14–16] using two assays. In the first assay, 200 μL of AF solution (1 mg/mL in isopropanol) or UV-filters (BP-3 at 50 μg/mL; 3-(4 methylbenzylidene)-camphor (MBC) at 10 μg/mL; octyl-methoxycinnamate (OMC) at 0.01 μL/mL and octocrylene (OCT) at 0.08 μL/mL) were transferred to a 96-well microplate. In the second assay, 100 μL of the fraction and 100 μL of UV-filters were mixed in wells of a 96-well microplate. Subsequently, spectrophotometric scanning was performed at wavelengths between 290 and 320 nm, with intervals of 5 nm, using μ-Quant microplate reader. The linearity of the reading ranges at each wavelength was previously checked with the calibration curves of UV-filters solutions at 10, 25, 30, 50 and 100 μg/mL and correlation coefficients higher than 0.99 were reached. The SPF was calculated according to the Mansur et al. [17]:

$$ \mathrm{SPF}=\mathrm{CF}\times \Sigma \left[\mathrm{E}\left(\uplambda \right)\times \mathrm{I}\left(\uplambda \right)\times \mathrm{AU}\left(\uplambda \right)\right] $$

in which E(λ) is the erythmogenic effect of the radiation, Ι(λ) the sunlight intensity and AU(λ) the absorbance. The values of E(λ) multiplied by I(λ) are normalized constants at each interval of 5 nm within the wavelength range measured and are given by Sayre et al. [18]. CF is a correction factor equal to 10.

### Fish embryo-larval toxicity (FET) test

Adult male and female zebrafish (*Danio rerio*) were obtained from a commercial supplier and kept in separate tanks (ethical approval UFG No. 102/2014). Fish culture and maintenance conditions were previously described by our group [19]. The fish embryo toxicity test with zebrafish was carried out according to OECD Test Guideline (TG) 236 [20] with some modifications such as test chambers (96-well plate) and volume to cover eggs (200 μL/well). Zebrafish eggs were collected approximately 30 min after natural mating, rinsed in water, and examined under a stereomicroscope (Bel Photonics STM PRO, Milano, Italy). Unfertilized or injured eggs were discarded. The fertilization success was checked, and only batches of eggs with fertilization rate above 90% were used. Twenty fertilized eggs per concentration were randomly selected and carefully distributed to a 24-well plate, filled with 2 mL of different concentrations of AF (0.01, 0.1, 1, 10 e 100 mg/L) or negative (NC; maintenance water) and positive controls (PC; 3,4-dichloroaniline at 4.0 mg/L). Four fertilized eggs were used as internal plate control (maintenance water) on each test and control groups. The test was performed in a climate chamber at 26 ± 1 °C and 12-h light. Neither food nor aeration was provided during the assays. Embryo development was assessed at 24, 48, 72, and 96-h post-fertilization using a stereomicroscope. The distinction between the normal and abnormal development of embryos was established according to the zebrafish development descriptions reported by Kimmel et al. [21]. Lethal (egg coagulation, no somite formation, non-detachment of the tail from yolk sac, and no heart beating) and sublethal (effects on the eye and body pigmentation, absorption of the yolk sac, hatching rate, swimming bladder inflation, otolith, presence of edemas and blood accumulation, tail deformities) parameters were observed and reported.

### Alginate encapsulation of HaCaT cells and spheroid formation

Human keratinocyte cells (HaCaT) were acquired from the Cell Bank of Rio de Janeiro (Rio de Janeiro, Brazil). Dulbecco’s Modified Eagle’s Medium (DMEM) (Life technologies, New York, USA) completed with 10% of fetal bovine serum (FBS) and 1% of antibiotic solution (100 IU/ mL penicillin to 100 μg/mL streptomycin, Life, USA) was used to grow the cells at 37 °C and 5% CO_2_ in humid atmosphere. The 3D cell culture was carried out according to Ferraz et al. [22, 23]. Briefly, after approximately 80% confluence in a monolayer culture, HaCaT cells were trypsinized and resuspended at a density of 5 × 10^5^ cells/mL in a 1.2% alginate MVM solution (Pronova, UP MVM, Novamatrix, Sandvika, Norway). Using a syringe attached to a 21-gauge needle, the mixture of alginate and cells was placed into a beaker containing 25 mL of 102 mM CaCl_2_ (Sigma, St. Louis, MO), forming alginate scaffolds containing approximately 20,000 cells within. The scaffolds were washed twice with 0.9% NaCl, once with complete medium, and then cultured in 6-well plates at 37 °C and 5% CO_2_ for 4 days with medium replaced all days until cell spheroids were formed. The details about the count of cells within the scaffold were previously described in detail by our group [23].

### Treatment of cells with residual aqueous fraction (AF)

About 2 × 10^4^ HaCaT cells grown in monolayers and aggregated into spheroids in 3D alginate scaffolds were seeded into each well of a sterile flat-bottomed 96-well plate, and incubated with 0.4 μg/mL to 10 mg/mL AF (diluted in DMEM supplemented with 10% FBS) for 24, 48 or 72-h. DMEM supplemented with 10% FBS was used as the negative control and 2% Triton-X100 as the positive control.

### WST-1 assay

The water-soluble tetrazolium salt assay (WST-1) was used to determine the number of viable cells after exposure to AF. Briefly, after treatment, the culture medium was replaced by 90 μL fresh culture medium and 10 μL WST-1 reagent (4-[3-(4-iodophenyl)-2-(4-nitrophenyl)-2H-5-tetrazolium]-1,3-benzene disulfonate) (Roche Co., South San Francisco, CA) and incubated at 37 °C and 5% CO_2_ for 3-h. The absorbance was then measured at 450 nm using a Polaris Microplate Reader (Celer, Brazil) according to the kit protocol. The intensity of the yellow color in the negative control wells was designated as 100% viability and all further comparisons were based upon this reference level.

### Lactate dehydrogenase (LDH) activity assay

After AF treatment, the integrity of the cell membrane was evaluated by measuring release of intracellular LDH which reduces NAD^+^ to NADH^+^/H^+^ by oxidation of lactate to pyruvate. Two hydrogen radicals released react with tetrazolium salt to yield a red formazan salt. The LDH cytotoxicity assay was carried out according to the manufacturer’s instructions (Roche, Mannheim, Germany). Briefly, 100 μL supernatant and 100 μL reaction mixture (freshly prepared) were transferred from each well of a 96-well flat-bottomed plate. The plates were incubated for 30 min at 20 °C in the dark and absorbance was measured at 492 nm using a Polaris Microplate Reader. Blank values indicating the absorbance of the LDH were subtracted from all samples. The percent cytotoxicity was calculated according to the kit protocol.

### In vitro 3T3 NRU phototoxicity assay

The experiment of in vitro 3T3 NRU assay was carried out according to OECD guideline [24]. The mouse fibroblast cell line, Balb/c 3T3, clone 31, was acquired from the Cell Bank of Rio de Janeiro (Rio de Janeiro, Brazil). The cells were cultivated in DMEM containing 10% fetal calf serum (FCB), 1% glutamine and 1% penicillin and streptomycin. About 1 × 10^4^ BALC/c cells per well were seeded in two 96-well microplates containing DMEM supplemented with 10% FCB and cultured overnight. Cells were then exposed to dilutions (7.81, 15.62, 31.25, 62.5, 125, 250, 500, 1000 μg/mL) of the AF and/or UV filters (BP-3, MBC, OMC and OCT) in Earle’s Balanced Salt Solution for 60 min. After the cells were irradiated at dose of 5 J/cm^2^ (1.7 mW/cm^2^) for 50 min using a UV Crosslinker (Ultra-violet Products Ltd., Upland, CA) model CL–1000 L (365 nm). After UV exposure, the test solution was replaced by fresh medium. Non-irradiated plate was kept at room temperature in a dark environment for 50 min. Cytotoxic effects were determined 24-h later by measuring the neutral red uptake (NRU). Neutral red (NR) solution (50 μg/mL) was added to the cell cultures (100 μL per well) and incubated for 3 h at 37 °C. The cells were then washed with PBS and the neutral red dye was extracted from the lysosomes using NR desorb solution (freshly prepared water + ethanol + acetic acid 49:50:1). Uptake of dye was measured spectrophotometrically at 540 nm using μ-Quant microplate reader. Tetracycline was used as a positive control (1.87–30 μg/mL under UVR and 100–1000 μg/mL without UVR). Sodium lauryl sulfate was used as a negative control (20–100 μg/mL). Photo Irritation Factor (PIF) and Mean Photo Effect (MPE) were calculated using Phototox 2.0 software. According to the OECD TG 432 [24], a test substance with a PIF < 2 or MPE < 0.1 is predicts as no phototoxic. A PIF > 2 and < 5 or MPE > 0.1 and < 0.15 is predicts as probable phototoxicity and a PIF > 5 or MPE > 0.15 predicts as positive phototoxicity.

### Statistical analysis

The significance of differences was calculated using one-way ANOVA and Dunnett’s post hoc test. In vitro FPS was analyzed by the *t*-Student test. The graphics and all statistical analyzed were performed using GraphPad Prism. All the experiments were carried out in triplicate and repeated twice. The data were expressed as mean ± standard deviation. *P* < 0.05 were considered statistically significant.

## Results

### UV–vis analysis

**Fig. 1:**
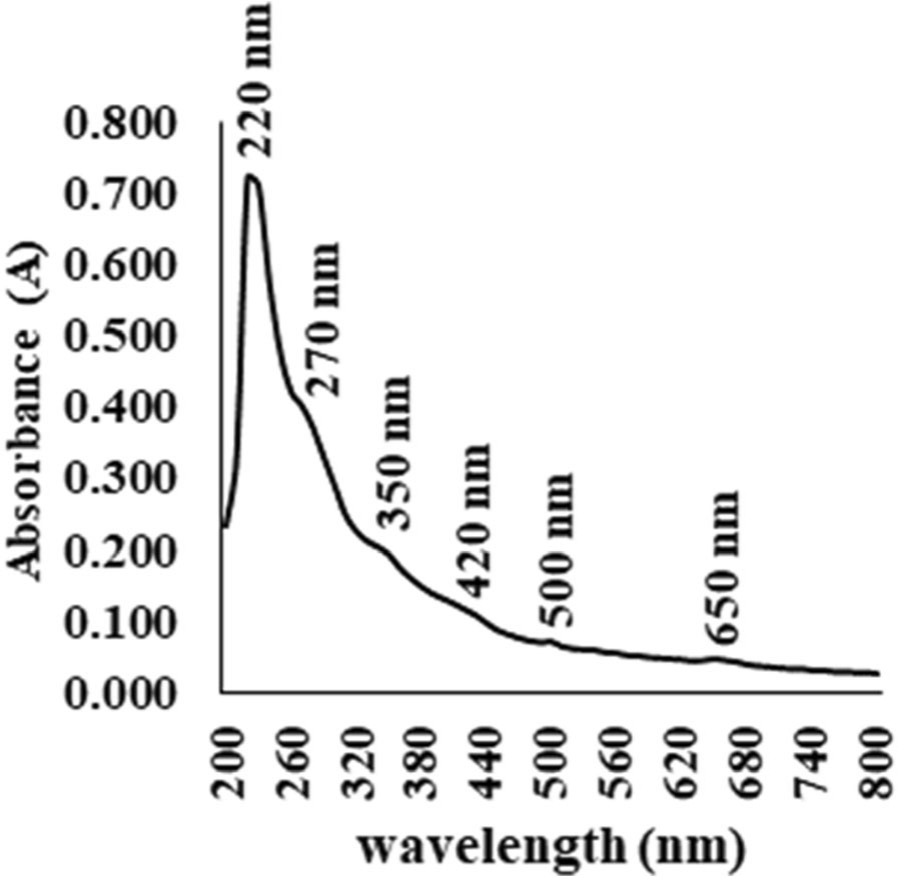
UV-VIS spectrum of the residual aqueous fraction (AF) from hydroalcoholic extract of the polar moss *Sanionia uncinata*

The absorption spectrum of AF at UV-visible light is shown in Fig. 1. This residual aqueous fraction absorbs in the spectral range of 200 to 700 nm, with maximum wavelength (λmax) at 220 nm. Other lesser intense absorption bands (of λmax 270, 350, 420, 500 and 650 nm) were also detected.

### In vitro SPF

**Fig. 2:**
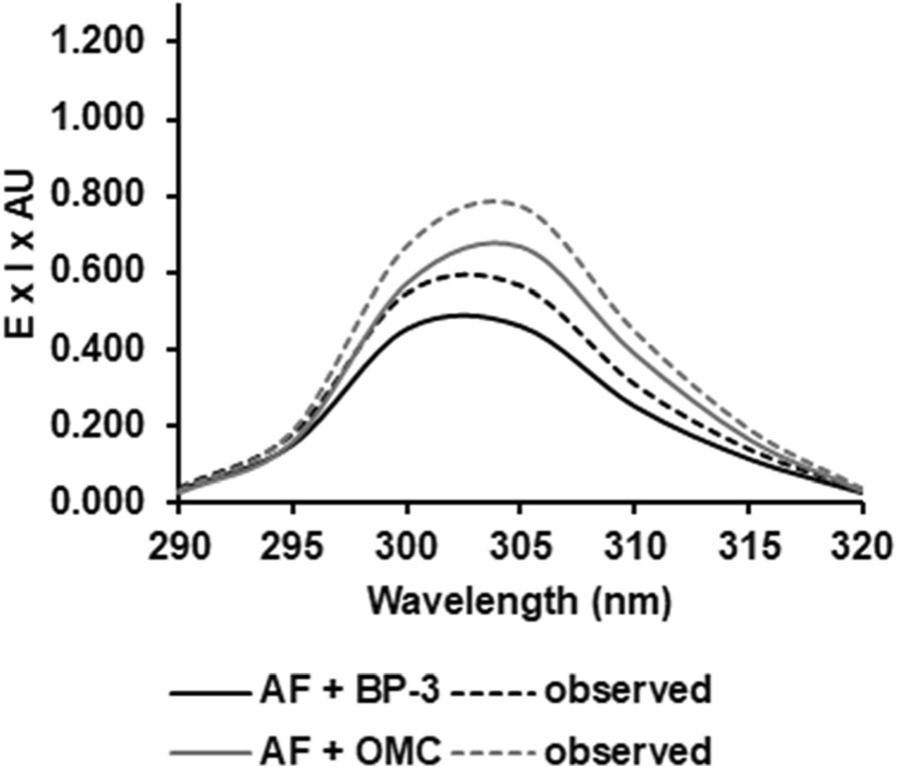
Relationship between erythmogenic effect of the radiation (E), sunlight intensity and (I) and absorbance (AU) with wavelength range. AF: aqueous fraction; BP-3: benzophenone-3; OMC: octyl-methoxycinnamate

The SPF values obtained at 2 mg/mL was 2.5 ± 0.3 (Table 1). Except for MBC and OCT that have an expected natural additive increasing on absorption intensity, the association of AF with BP-3 and OMC led to a significant higher increase (*P* < 0.05, *t*-Student) of SPF values: 12.3 ± 0.9 for BP-3 increased to 18.0 ± 1.2, equivalent to ~ 3-fold increasing; 17.6 ± 0.8 for OMC increased to 24.5 ± 0.7 equivalent to ~ 4-fold increasing. The significant synergic effect between the AF and these UV-filters can be seen in the Fig. 2, in which are represented the calculated E x I x AU curves of AF + BP-3 and AF + OMC mixtures determined from observed absorption and by predicting if only additive absorption would be presented.

**Table 1:** Absorbances of residual AF from hydroalcoholic extract of *Sanionia uncinata* and the spectrophotometrically calculated SPF

(nm)	AF	AF plus UV-filters			
		BP-3	MBC	OMC	OCT
290	0.33 ± 0.03	2.40 ± 0.14	2.57 ± 0.04	2.32 ± 0.07	2.59 ± 0.12
295	0.30 ± 0.03	2.16 ± 0.14	2.72 ± 0.04	2.41 ± 0.07	2.67 ± 0.12
300	0.27 ± 0.03	1.90 ± 0.13	2.75 ± 0.04	2.44 ± 0.07	2.70 ± 0.12
305	0.24 ± 0.02	1.72 ± 0.12	2.64 ± 0.03	2.47 ± 0.07	2.66 ± 0.12
310	0.22 ± 0.02	1.64 ± 0.11	2.45 ± 0.03	2.52 ± 0.07	2.60 ± 0.12
315	0.20 ± 0.02	1.63 ± 0.10	2.13 ± 0.03	2.40 ± 0.07	2.45 ± 0.12
320	0.19 ± 0.02	1.63 ± 0.10	1.70 ± 0.02	2.14 ± 0.06	2.25 ± 0.11
SPF	2.5 ± 0.3	18.0 ± 1.2^*^	25.8 ± 0.3	24.5 ± 0.7^*^	26.4 ± 1.2

Sun Protection Factor (SPF) of UV-filters: benzophenone-3, BP-3 (12.3 ± 0.9); 3-(4-methylbenzylidene)-camphor MBC (24.8 ± 1.4); octyl-methoxycinnamate, OMC (17.6 ± 0.8); and octocrylene, OCT (22.6 ± 0.1); AF: aqueous fraction; ^*^Synergistic effect compared to the respective UV-filters (*P* < 0.05, *t*-Student)

### Fish embryo-larval acute toxicity test

**Fig. 3:**
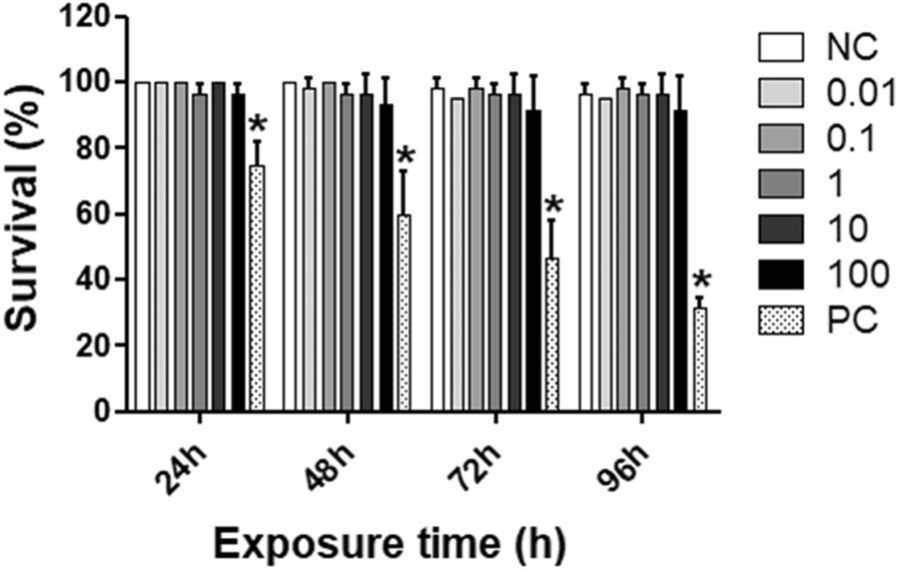
Percentage of survival on zebrafish early-life stage after 24, 48, 72, and 96-h of aqueous fraction (AF) from extract of the Antarctic moss *Sanionia uncinata* exposure (0.01 to 100 mg/L). Bars represent the mean ± standard deviation. *P < 0.001 statistically different from the respective negative control. NC = negative control (maintenance water); PC = positive control (3,4-dichloroaniline at 4.5 mg/L)

**Fig. 4:**
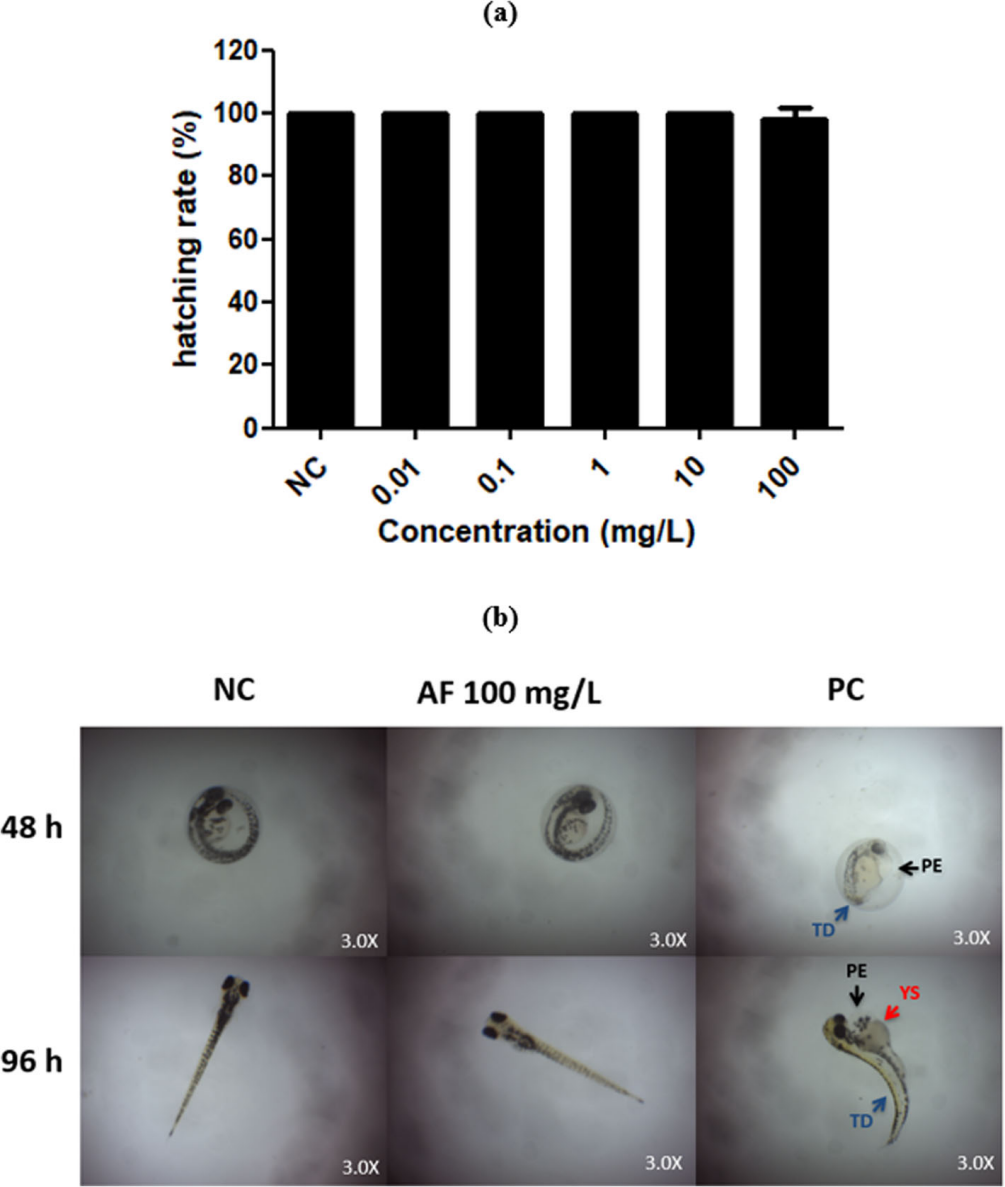
**a** Effects of the aqueous fraction (AF) from extract of the Antarctic moss *Sanionia uncinata* on hatching success of zebrafish embryos. Bars represent the mean ± standard deviation. The values do not differ significantly from each other (*P* > 0.001). **b** Representative photomicrographs of the absence of abnormalities in zebrafish at early life-stage induced by AF during 96-h of exposure. NC: negative control; PC: positive control; PE: pericardial edema; tail deformation (TD) and YS: delayed yolk sac absorption

The embryotoxicity of different concentrations (0.01 to 100 mg/L) of AF processes on zebrafish early-life stage was recorded at 24, 48, 72 and 96-h. No significant lethal or sublethal effect was observed for AF during 96-h of exposure. Figure 3 shows survival rate on zebrafish early-life stages after 24, 48, 72 and 96-h of exposure. Besides, hatching rate of embryos was not affected by AF until 96-h, as well as did not cause any significant malformations in zebrafish until 96-h of exposure compared to negative control that exhibited a normal embryonic development (Fig. 4a and b). Thus, it seems to have no embryotoxic potential in this concentration range.

### WST-1 and LDH assays – monolayers and 3D cells

**Fig. 5:**
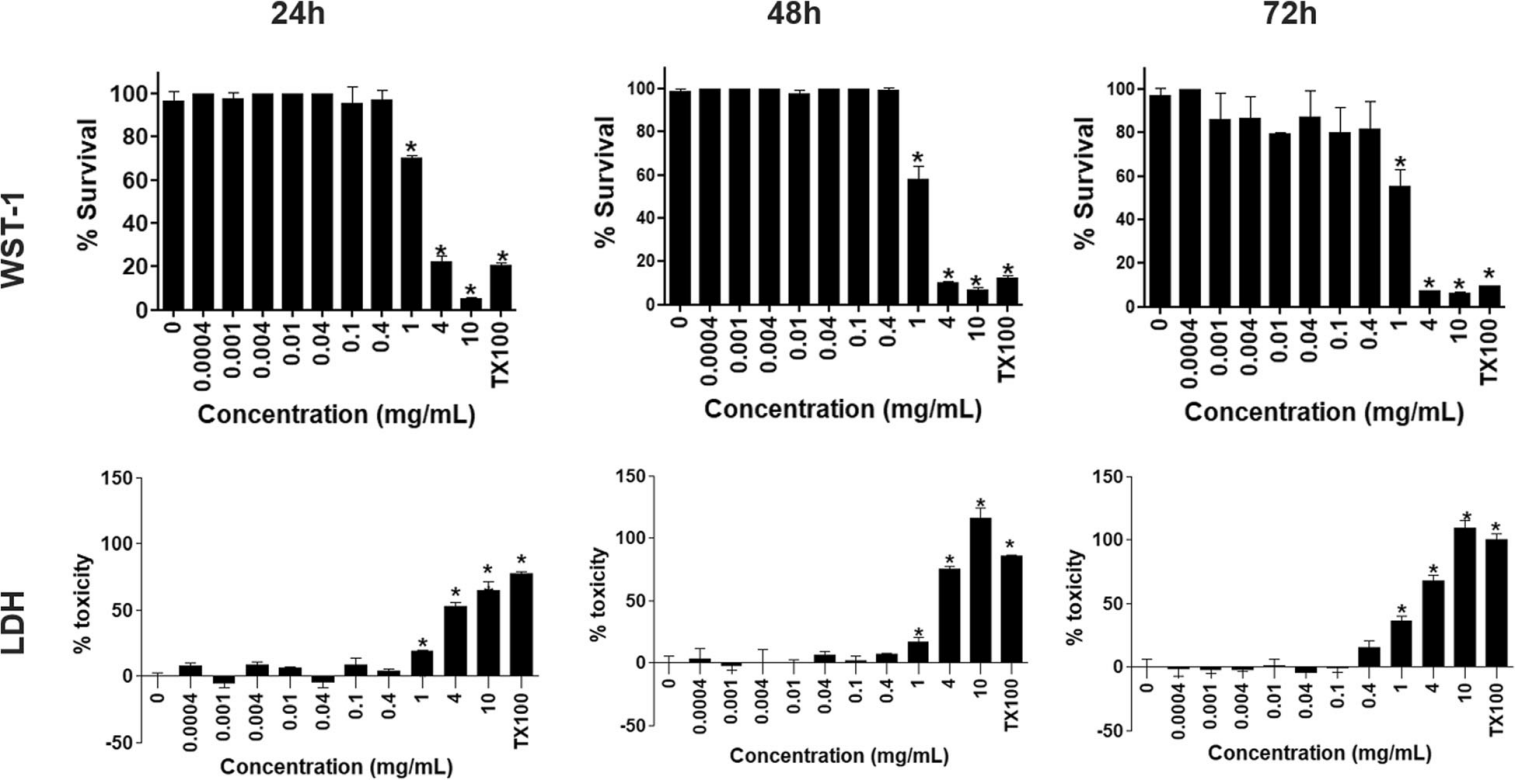
Effects of aqueous fraction (AF) from extract of the Antarctic moss *Sanionia uncinata* on viability by mitochondrial activity (WST-1) or cell membrane integrity (LDH) of HaCaT cell cultured in monolayers after 24, 48, and 72-h of exposure. The asterisk indicates significant differences at *P* < 0.05 as related to the negative control. TX100: Triton X-100 (5%) as positive control

**Fig. 6:**
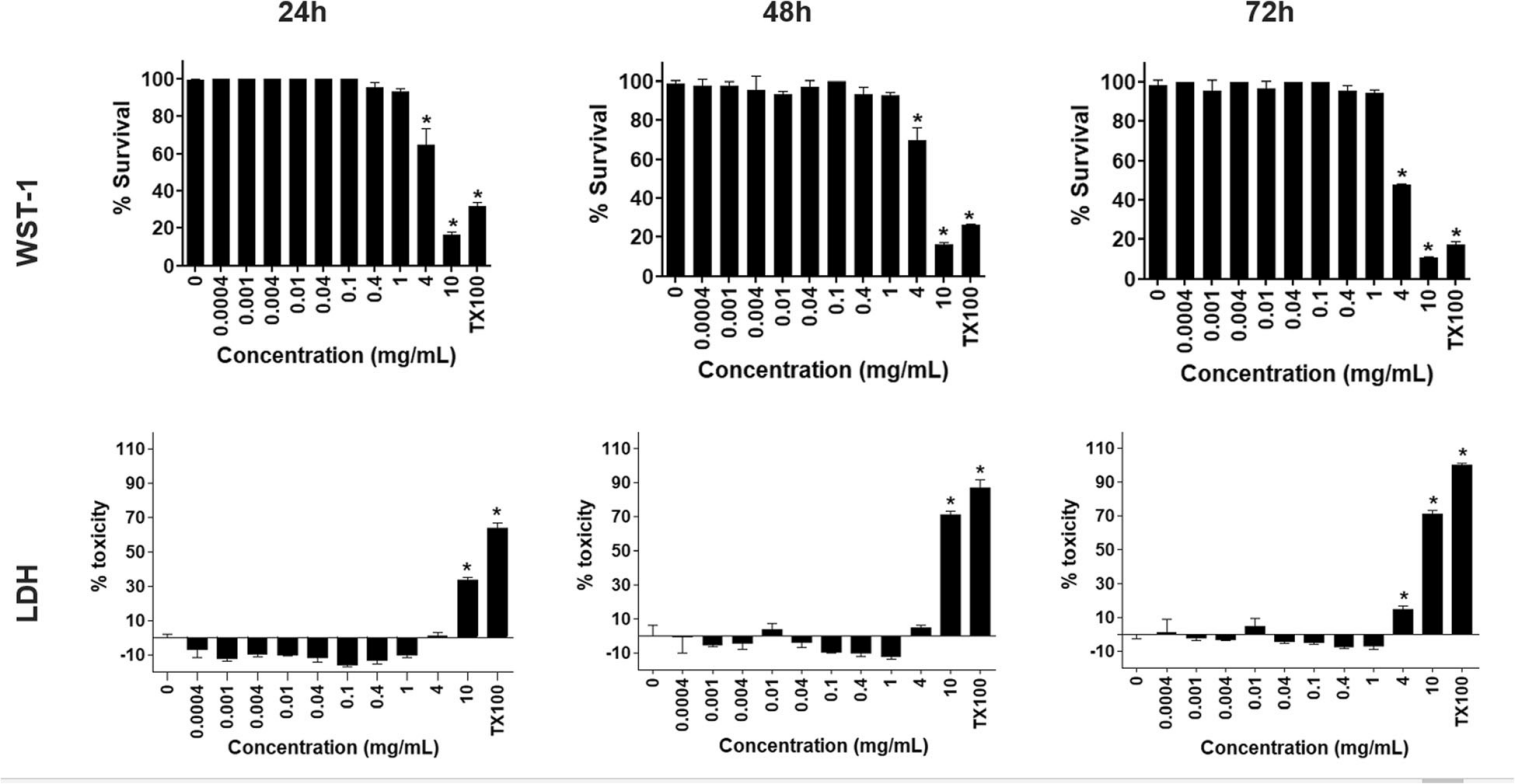
Effects of aqueous fraction (AF) from extract of the Antarctic moss *Sanionia uncinata* on viability by mitochondrial activity (WST-1) or cell membrane integrity (LDH) of 3D cultured HaCaT cell after 24, 48, and 72-h of exposure. The asterisk indicates significant differences at *P* < 0.05 as related to the negative control. TX100: Triton X-100 (5%) as positive control

Figure 5 shows the influence of AF on the viability of mitochondrial dehydrogenase activity and on cell membrane integrity of HaCaT keratinocyte cell line cultured in monolayers after 24, 48 and 72-h of incubation. In monolayers, the HaCaT cell viability evaluated by WST-1 was above 70% by ≤0.4 mg AF/mL after 48 and 72-h exposure, whereas was ≤1 mg AF/mL after 24-h exposure. The LDH assay showed that the cell viability was above 70% by ≤0.4 mg AF/mL even after 72-h exposure, but was ≤1 mg/mL after 24 and 48-h exposure (Fig. 5). In 3D cell culture (Fig. 6), an increased cell resistance to toxicity was observed, because viability of HaCaT cells by WST-1 and LDH was above ~ 90% when using ≤1 and 4 mg AF/mL, respectively, in all exposure times.

### In vitro 3T3 NRU assay

There is consensus in the classification of phototoxic potential of AF and its association with different UV-filters using either the PIF or MPE grading schemes in the 3T3 NRU testing results (Table 2). AF was considered no phototoxic, since it presented mean PIF of 1.089 and mean MPE of 0.035. In combinations with UV-filters the AF did not also present any phototoxic potential (PIF < 2 and MPE < 0.1).

**Table 2:** Phototoxicity of isolated residual AF and its association with UV-filters (BP-3, OMC, OCT, MBC)

Substance	PIF	MPE
AF	1.089	0.035
AF + BP-3	0.736	−0.020
AF + MBC	1.377	−0.046
AF + OMC	1.843	0.087
AF + OCT	0.142	−0.321
SLS (negative control)	0.856	−0.030
Tetracycline (positive control)	40.481^a^	0.539^a^

*AF* aqueous fraction, *BP-3* benzophenone-3, *OMC* octyl-methoxycinnamate, *OCT* octocrylene, *MBC* and 3-(4-methylbenzylidene)-camphor, *SLS* sodium lauryl sulfate, *PIF* Photo-Irritation-Factor, *MPE* Mean Photo Effect, BP-3 (PIF: 1.798, MPE: 0.152); OMC (PIF: 1.741, MPE: 0.105); OCT (PIF: 0.618, MPE: -0.034); MBC (PIF: 0.112, MPE: -0.133)

^a^classified as phototoxic

## Discussion

Our research group has previously reported important photoprotective properties of extracts from Antarctic moss *S. uncinata*, as well as its toxicological activities of its organic extracts. We have also demonstrated positive correlation between protective effect against UVR and phenolic compound present in extracts. Water-extractable compounds seem to contribute on photoprotection of this Antarctic moss [10, 13, 14]. Given the above context, the goal of this paper is to explore the potential photoprotective, cytotoxic and embryotoxic effects of residual AF from the polar moss *S. uncinata*. This fraction, was obtained through the liquid-liquid partitioning of the HE of this moss. The UV-visible spectrum showed that AF absorbs in the UV-visible light spectra. AF exhibits UV peaks absorption (200–420 nm) similar to the major flavonoids such as aurones, chalcones, flavones and flavanols [25]. These results corroborate with the data obtained previously with HE using HPLC-DAD, in which flavonoids, including these classes, were characterized [14]. In plants, the flavonoids constitute an enormous class of phenolic natural products. Present in most plant tissues flavonoids such as quercetin derivatives and other dihydroxy B-ring substituted flavonoids are able of UV absorbing and scavenge UV-generated ROS to protecting plants from UV radiation of sun [26, 27]. The λmax in visible spectrum (> 400 nm) shown by the fraction can be from traces of chlorophylls (420 and 650 nm by additive absorption of chlorophyll a and chlorophyll b) and carotenoids (500 nm), both classes remaining of partition process since high content of these compound in mosses is known [12, 28].

In vitro SPF has been applied to be linear, precise, accurate, specific and robust [15]. In order to evaluated the photoprotection efficacy of AF by absorbing UVR at the erythema-inductor region, the in vitro SPF was determined. Despite the low value of SPF obtained by AF alone, the SPF values of the UV-filters BP-3 and OMC, increased significantly (*P* < 0.05, *t*-Student) when associated with AF. Recently our research group observed similar features for the different extracts of the Antarctic moss *S. uncinata*, including HE [14]. This suggests that the more polar character of the constituents of the HE leave to an increased intensity of band K (λmax 260 nm, π-conjugated transition), causing the hyperchromic effect observed [29]. It is well documented that flavonoids and other phenol derivatives can also act in synergy with vitamin C/vitamin E, for example [30]. Natural compounds such as polyphenols can be more effective as sunscreens than synthetic chemicals, due to their long-term beneficial effects mainly against free radicals. In addition, today there is trend to develop high UV protection sunscreens using low concentrations of synthetic products [31], since chemical sunscreens have been reported for causing adverse secondary effects in human [7]. The same are known that UV-filters have the potential to cause abnormalities in development of zebrafish embryos [32]. In this sense, the embryotoxicity of the AF was investigated using the zebrafish as an organism model, by assessing their lethal and sublethal endpoints. The zebrafish early life-stage is an important model because offers a complex and multicellular system integrating the interaction of several tissues and differentiation processes [33] that permit drug screening and embryotoxicity assessment simultaneously [34]. Besides that, the transparency embryo and the development outside of the mother allow scoring of embryotoxic effects easily [34]. Our results showed that AF was not toxic to zebrafish early life stage. Besides, AF did not induce significantly lethal effects and malformations in zebrafish development and this fraction was not able to induce delay or embryo hatching inhibition. Thus, it seems AF have no embryotoxic potential. The gold standard for human application will remain mammals. However, employing zebrafish embryos at an early drug development stage could possibly reduce cost of new drugs significantly and will certainly provide an ethically more acceptable alternative to traditional testing [34].

Regarding the cytotoxic evaluation, the AF until 0.4 mg/mL did not significantly affect the mitochondrial dehydrogenase activity, as detected by the WST-1 assay, in cultured in monolayers HaCaT cells. Similar results were obtained by the cell membrane integrity, as shown by the LDH activity assay. In 3D cell culture, an increased cell resistance to toxicity was observed, since cell viability of HaCaT by WST-1 and LDH measurement was above 90% when using ≤1 and 4 mg/mL, respectively, in all exposure times. Comparatively to conventional 2D cultures, 3D cell culture reproduces better the tissue architecture in vivo, does forecast organ-specific toxicity and emulates more closely the biochemistry and mechanics of the microenvironment in tissues [23]. When exposed to a xenobiotic, the behavior of cells is related to tissue architecture. Likely, the difference in behavior between monolayer and in 3D cells may be associated with the spatial geometry and distribution of cells in both systems [35]. In spheroid cultures, grown within alginate scaffolds, as used in this work, HaCaT cells can secrete extracellular matrix and interact with cells from their original microenvironment [36]. Thus, this type of cell culture system might simulate in vivo processes, such as cell–cell contact, altered metabolism, variations in the cell cycle, and diffusion of nutrients, oxygen or xenobiotics [37]. Thus, the increased cell resistance to toxicity may be attributed to cell arrangement.

The 3T3 NRU phototoxicity assay, used as endpoint for phototoxic hazard, has showed high sensitivity and specificity [38]. It has been validated and accepted for regulatory purposes, obtaining a correlation of in vivo and in vitro results above 95% [39, 40]. AF demonstrated values of PIF < 2 and MPE < 0.1 (no phototoxicity), even though in combinations containing UV-filters such as BP-3, MBC, OMC and OCT.

## Conclusions

The current study has shown that the Antarctic moss *S. uncinata* contains important constituents for its protection against photobiological damage and the associated oxidative stresses. The compounds present in *S. uncinata* could have great potential for health, cosmetic and dermatological applications. The UV–visible analysis induced optical absorption spectra recorded in the UV-visible light spectra. The fraction showed enhanced SPF of BP-3 and OMC, indicating that its constituents could be considered as attractive candidates for protection against UV-induced erythema formation. Additionally, the AF did not induce phototoxic effects, even in combination with UV-filters. Despite cytotoxicity has been detected in the HaCaT cell cultured in monolayers, an increased cell resistance to toxicity was observed in 3D cell culture, suggesting that in this system, that more closely resembles in vivo cell growth, higher concentrations can be used without damage to the cell. Furthermore, the AF did not induce acute toxicity on zebrafish embryos, suggesting no embryotoxic potential. Therefore, the present study shows that residual AF presents a good potential for a skin photoprotection against UV-radiation.

## Data Availability

The datasets generated and/or analyzed during the current study are available with the corresponding author on reasonable request.

## Abbreviations

2D: Two dimensions
3D: Three dimensions
AF: Aqueous fraction
AU(λ): Absorbance
Bf: *n-*butanol fraction
BP-3: Benzophenone-3
CF: Correction factor
Df: Dichloromethane fraction
DMEM: Dulbecco’s Modified Eagle’s Medium
E(λ): Erythmogenic effect
EE: Ethanolic extract
Ef: Ethyl acetate fraction
FBS: Fetal bovine serum
FCB: Fetal calf serum
HE: Hydroethanolic extract
Hf: *n-*hexane fraction
LDH: Lactate dehydrogenase
MBC: 3-(4-methylbenzylidene)-camphor
ME: Methanolic extract
MPE: Mean Photo Effect
NR: Neutral red
NRU: Neutral red uptake
OCT: Octocrylene
OMC: Octyl-methoxycinnamate
PIF: Photo-Irritation-Factor
ROS: Reactive oxygen species
SLS: Sodium lauryl sulfate
SPF: Sun protection factor
UVA: Ultraviolet A
UVB: Ultraviolet B
UVC: Ultraviolet C
UVR: Ultraviolet radiation
WST-1: Water-soluble tetrazolium salt
Ι(λ): Sunlight intensity
